# Improved DNase-seq protocol facilitates high resolution mapping of DNase I hypersensitive sites in roots in *Arabidopsis thaliana*

**DOI:** 10.1186/s13007-015-0087-1

**Published:** 2015-09-04

**Authors:** Jason S. Cumbie, Sergei A. Filichkin, Molly Megraw

**Affiliations:** Department of Botany and Plant Pathology, Oregon State University, Corvallis, OR 97331 USA; Department of Electrical Engineering and Computer Science, Oregon State University, Corvallis, OR 97331 USA; Center for Genome Research and Biocomputing, Oregon State University, 2082 Cordley Hall, Corvallis, OR 97331 USA

**Keywords:** DNase-seq, DNase I hypersensitive sites, Open chromatin, Arabidopsis, Roots, Nuclei

## Abstract

**Background:**

Identifying *cis*-regulatory elements is critical in understanding the direct and indirect regulatory mechanisms of gene expression. Current approaches include DNase-seq, a technique that combines sensitivity to the nonspecific endonuclease DNase I with high throughput sequencing to identify regions of regulatory DNA on a genome-wide scale. While this method was originally developed for human cell lines, later adaptations made the processing of plant tissues possible. Challenges still remain in processing recalcitrant tissues that have low DNA content.

**Results:**

By removing steps requiring the use of gel agarose plugs in DNase-seq, we were able to significantly reduce the time required to perform the protocol by at least 2 days, while also making possible the processing of difficult plant tissues. We refer to this simplified protocol as DNase I SIM (for simplified in-nucleus method). We were able to successfully create DNase-seq libraries for both leaf and root tissues in *Arabidopsis* using DNase I SIM.

**Conclusion:**

This protocol simplifies and facilitates generation of DNase-seq libraries from plant tissues for high resolution mapping of DNase I hypersensitive sites.

**Electronic supplementary material:**

The online version of this article (doi:10.1186/s13007-015-0087-1) contains supplementary material, which is available to authorized users.

## Background

*Cis*-regulatory elements (CREs) are short DNA sequences which are used by regulatory proteins such as transcription factors (TFs) to control the expression of genes [1]. Because these elements need to be physically accessible to their respective regulatory proteins, they are often found in regions of the genome known as ‘open chromatin’ that are either unbound by or depleted of nucleosomes [1]. Binding of regulatory proteins to their target DNA sequences can cause dynamic chromatin rearrangements resulting in displacement of nucleosomes in the regions of accessible chromatin (reviewed in [1, 2]).

Chromatin accessibility and the effects of chromatin structure modifications on gene transcription can be assessed directly and indirectly. Direct chromatin accessibility assays include Formaldehyde-Assisted Isolation of Regulatory Elements (FAIRE). FAIRE-seq [3] is a relatively simple method for probing nucleosome-depleted regions of a genome. However, a high level of background noise in the output data limits its resolution and value [3]. Due to the lack of tightly bound histone proteins, regions of open chromatin are more readily digested by endonucleases such as micrococcal nuclease (MNase) and deoxyribonuclease I (DNase I). MNase is a low specificity endo-exonuclease that digests single-stranded, double-stranded, circular, and linear DNA. In MNase-seq experiments (commonly referred to as a nucleosome occupancy assay), mononucleosomes are extracted by MNase digestions of formaldehyde-crosslinked chromatin [4]. The nucleosomal population is subsequently subjected to next generation sequencing (NGS), and nucleosome positioning is then deduced from the NGS read counts across the genome. Thus, MNase-seq is a method of choice for assessing genome-wide nucleosome positioning. It can also provide limited information on TF occupancy in different cell types [5]. Drawbacks of the MNase-seq method are that it requires a large number of cells and meticulous enzymatic titrations for reproducible evaluation across samples. In addition, MNase has been shown to have a bias toward AT-cleavage specificity and comparisons between different experiments may vary significantly. In contrast, DNase I is a double stranded DNA-specific endonuclease that releases accessible chromatin by preferentially digesting nucleosome-free genomic regions categorized as DNase I hypersensitive sites (DHSs). Using DNase I digestions of intact nuclei in conjunction with NGS, known as DNase-seq, allowed for genome-wide identification of DHSs with unmatched specificity, sensitivity, and throughput [6]. The improved quality of NGS data has made DNase-seq a preferred method of choice for probing chromatin accessibility in general, and TF occupancy in particular [6, 7].

DHSs have been shown to be strongly associated with CREs [8, 9]. While initial studies using DNase-seq were performed in human cell lines [6], DNase-seq was later adapted to plant tissues, with the first DNase-seq experiments occurring in rice seedling and callus tissue [8] and in *Arabidopsis thaliana* seedling and flower tissues [9]. A critical step in preparing DNase-seq libraries requires isolating intact nuclei. The isolation of nuclei in plants is especially challenging due to the existence of the cell wall. Removal of the rigid cell wall and additional cellular debris requires an extensive amount of additional time and added steps to ensure that nuclei are not lysed in the process. The susceptibility of DNA to mechanical shearing must be carefully avoided to ensure that DNase I digestion can occur under optimal conditions, and that background noise due to spurious DNA fragments is not introduced in down-stream analyses. The latter of these challenges was addressed by introducing low-melt gel agarose plugs during DNase I digestion and T4 DNA polymerase blunt end repair to stabilize high molecular weight DNA in mammalian cell lines [6]. Further adaptations to this protocol added a cell wall removal step [10]. Because of the extensive molecular processing steps required in DNase-seq, tissues that are more resistant to homogenization and that have fewer cells per gram of tissue isolated will be prohibitively challenging to examine. To address this difficulty, here we present a simplified DNase-seq protocol in plants that bypasses the use of low-melt gel agarose plugs. In this protocol, DNA end repair by T4 DNA polymerase is performed directly in nuclei, thus we refer to this simplified protocol as DNase I SIM (for simplified in-nucleus method).

Recently, other protocols such as DNase-Flash [11] have been successfully adapted using the INTACT system [12] for use with biotinylated nuclei obtained from transgenic *Arabidopsis* lines [13]. Where INTACT lines are available, the labor-saving ATAC-seq approach [14] that uses hyperactive Tn5 transposase to characterize DNA accessibility could also potentially be adapted to plant tissues, though output signal resolution in comparison to DNase-seq is still unclear. In this study, we have developed a purification and sequencing preparation that makes plant tissue studies using the original DNase-seq approach [6] feasible, even in recalcitrant tissues such as plant roots. We have successfully used the DNase I SIM protocol in *A. thaliana* leaf and root tissue, providing the very first DHS map in non-transgenic whole root tissue. This protocol greatly facilitates DHS sequencing in cases where an affinity purification system is not available. DNase I SIM thus provides an option that may be particularly desirable for DNase-seq studies in crop species where tissue is abundant but development of transgenic lines is impractical.

## Results

### DNase I SIM protocol allows isolation and digestion of nuclei from leaf and root tissue in substantially reduced time

The past use of low-melting agarose plugs in combination with a more vigorous nuclei isolation protocol [6, 10] made it possible to analyze DHSs in leaf and flower tissue in *Arabidopsis* and seedling and callus tissue in rice [8, 9]. However, we found that we were unable to produce sufficient quantities of DNAse I digested DNA for NextGen sequencing using a similar version of this protocol when processing *Arabidopsis* root tissue samples. A possible reason for the low DNA yield was a particularly high content of the cell debris (including broken root hairs) that co-purified with root nuclei. The enormous required volume of preparations was prohibitive for the embedding of sufficient amounts of nuclei into the constricted volume of a PFGE agarose plug. As a result, visualization of the digested DNA was difficult to monitor using PFGE. In addition, scaling up the number of plugs to achieve higher yield required a sharp increase in the amounts of the T4 polymerase in order to polish DNA ends. Thus, the usage of agarose plugs made the protocol time consuming, labor-intensive, and less predictable.

**Fig. 1:**
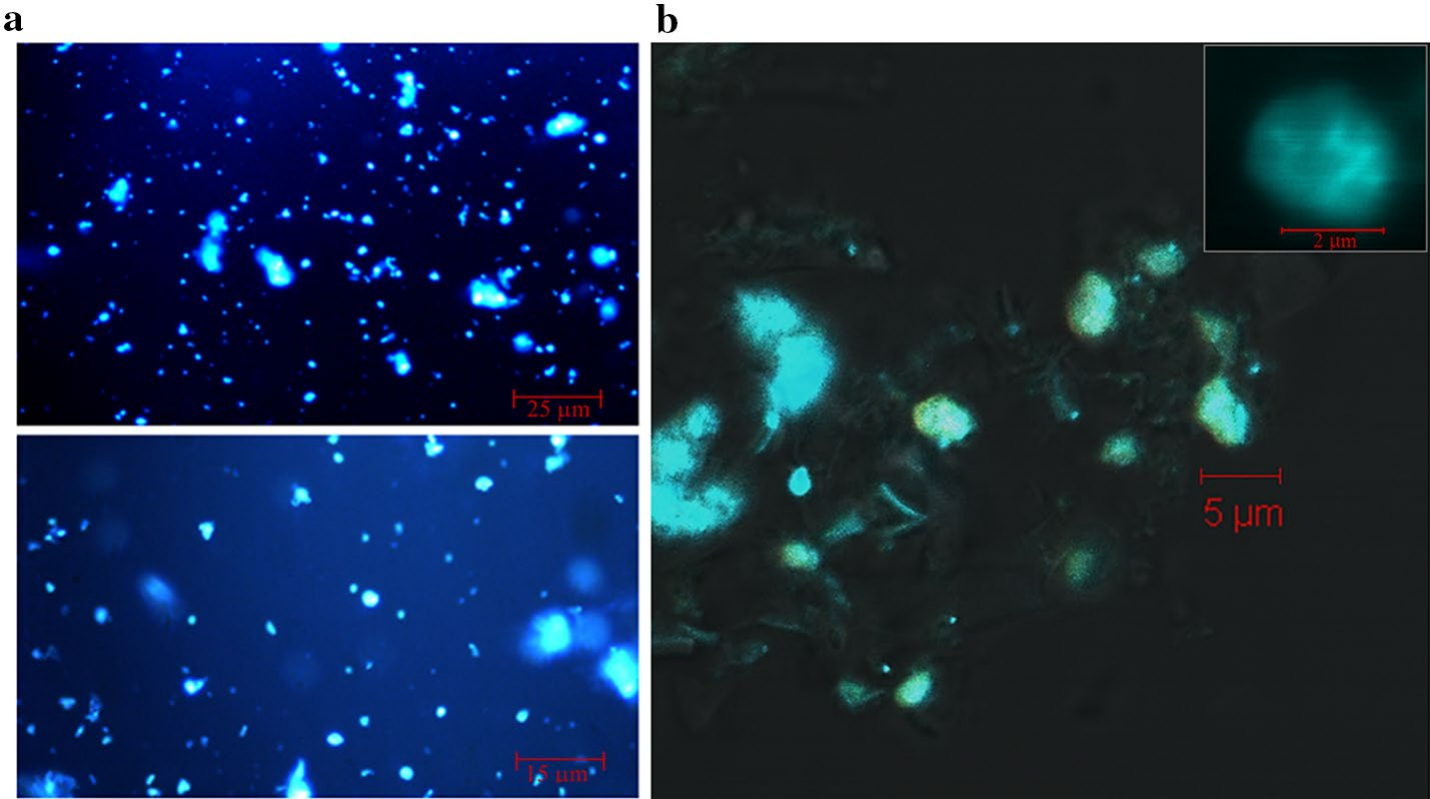
Intact nuclei from *Arabidopsis* roots. Nuclei prepared using optimized protocol and Percoll density gradients were stained with DAPI and observed using fluorescence microscopy and UV-light (**a**) or confocal microscopy (**b**) according to standard procedures

To circumvent these difficulties, we introduced three important changes to the previous protocol. First, an additional step of nuclei purification in Percoll gradients was added prior to DNAse I digestion in order to remove cellular debris and starch granules more efficiently. Second, DNA end polishing by T4 DNA polymerase was performed directly in the nuclei following DNAse I digestion. Finally, the use of agarose plugs was bypassed completely. Altogether, these modifications greatly simplified as well as increased speed and throughput of the protocol for DNase-seq library construction. Previously, T4 DNA polymerase was added only after nuclei were embedded into low-melt agarose and lysed [6, 10]. During protocol development, two critical observations allowed us to circumvent agarose plug usage. First, Percoll gradient-purified nuclei remained mostly intact after subsequent steps that terminate the DNase I digestion (e.g. EDTA treatment). Second, T4 DNA polymerase can be used to polish DNase I digested DNA ends directly in intact nuclei. The presence of intact nuclei during purification, DNase I digestion, and T4 DNA end polishing was monitored using DAPI staining and confocal microscopy (Fig. 1). These improvements simplified the protocol and resulted in a reduction of at least 2 days in the overall time required for DNase-seq library preparation. This modified protocol, DNase I SIM, was successfully used for DHS mapping in both root and leaf tissues of *Arabidopsis*.

### DNase I SIM protocol data validation

In order to ensure that the modifications made to the original DNase-seq protocol did not change the nature of the data produced, we compared our leaf data to published leaf data in *A. thaliana* [9]. We made these comparisons using three separate approaches that examined averaged DHS distribution genome-wide, across all genes, and a direct DHS-to-DHS comparison for identifying commonalities and differences for individual genes in all data sets analyzed. These same analyses were carried out using our data from root tissue. It is important to note that sequencing our leaf sample on the HiSeq-2000 generated nearly two-to-three times as many reads as had been previously published (100 × 10^6^ compared to 46 × 10^6^). To account for this difference in read depth, we provide separate analyses in which we randomly subsampled DNase I SIM data to a comparable read depth to provide the most direct comparisons. For these analyses our data is marked as “normalized”.

#### DHS genome distribution is depleted in centromeric and peri-centromic regions of the chromosome

**Fig. 2:**
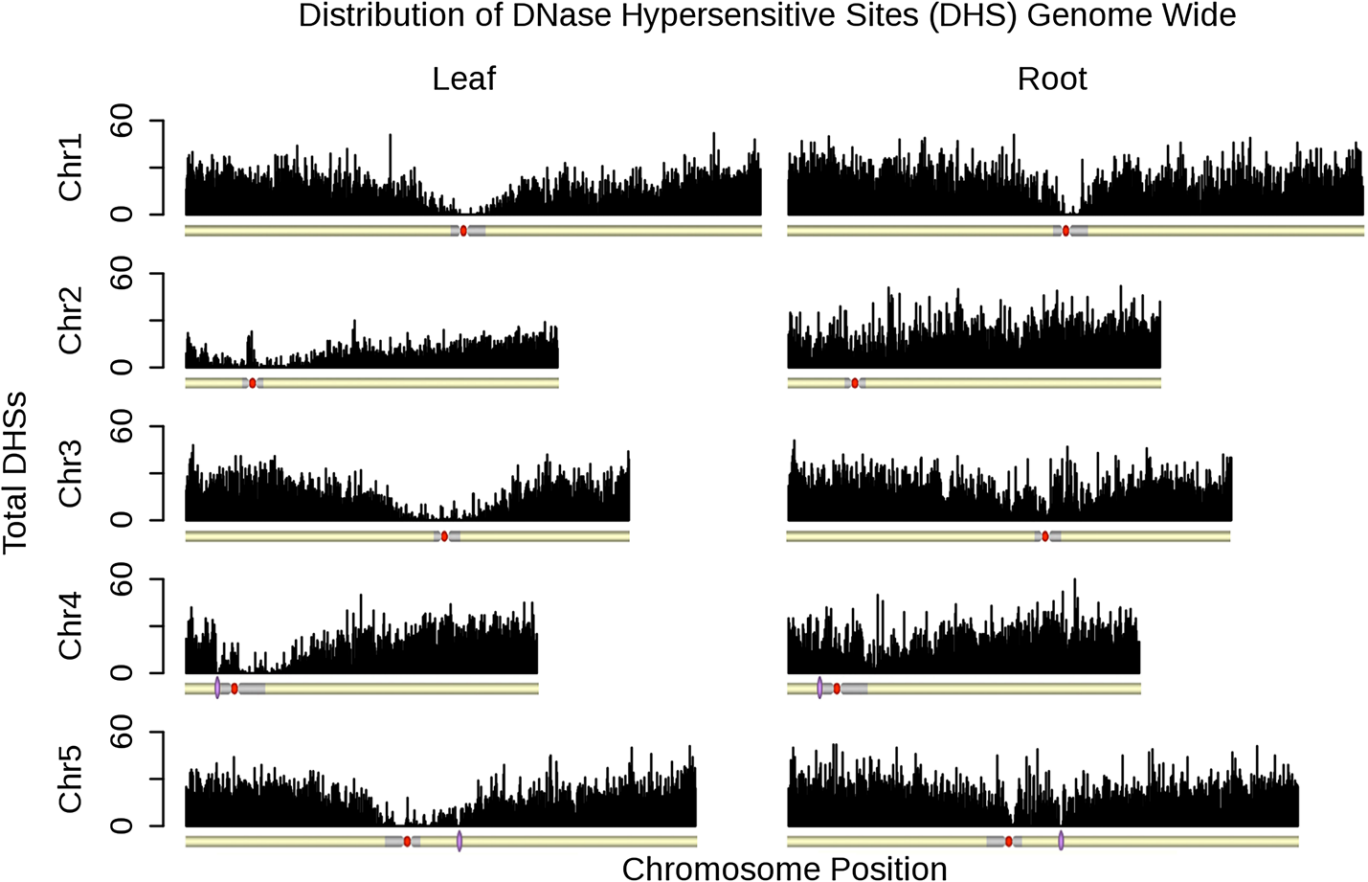
Distribution of DNase hypersensitive sites along *Arabidopsis* chromosomes. Approximate boundaries of *Arabidopsis* centromeres [16] are shown in *gray*. Non-sequenced centromeric gaps are indicated by *red circles*. Positions of heterochromatic knobs are denoted by *violet ellipses*

We produced DHS maps of our leaf control and root data using the F-Seq software package [15]. To ensure that the two data sets were directly comparable, we re-analyzed previously published data in *Arabidopsis* [9] using the most recent version of F-Seq, which was used for all data in our DHS comparisons. To map the genome-wide distribution of DHSs, we divided each chromosome into equal length bins (see “Methods”), and then enumerated the number of DHSs found within each bin for each chromosome. The distribution of DHSs along *Arabidopsis* chromosomes showed that centromeres had in general a lower density of open chromatin both in our DNase I SIM leaf and root data sets, as well as in the re-analyzed previously published leaf data [9] (Fig. 2; Additional file 1). These commonalities were also present when comparing our normalized leaf data set to previously published data sets (Additional file 2). This result was consistent with lower density of the expressed loci across centromeric regions [16].

#### DHS distribution on average localizes to promoter and transcriptional termination regions

**Fig. 3:**
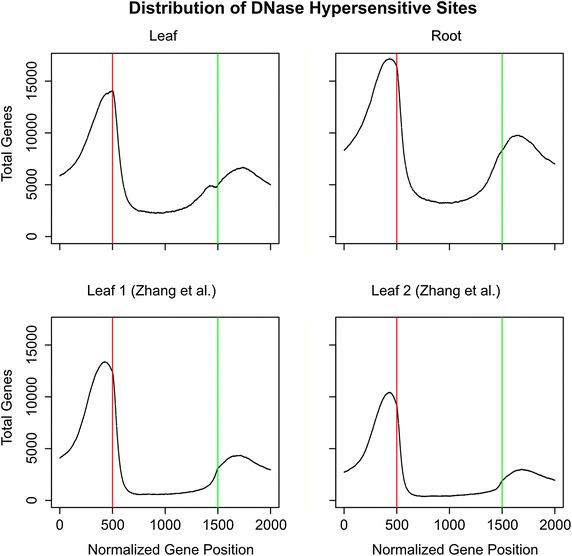
Distribution of DNase hypersensitive sites across genes. DHSs across our leaf and root samples (*top left* and *top right*) and both leaf replicates using previously published re-analyzed data [9] (*bottom left* and *bottom right*). The x-axis represents the normalized gene length, with positions 1–500 indicating the first 500 bp upstream of the TSS, and the red line indicating the TSS. Positions 501–1500 indicate the gene body, the *green line* indicates transcription termination. Positions 1501–2000 indicate the 500 bp downstream of the gene end

To generate a comparison of DHS gene localization, we generated a matrix with each gene represented by a separate row, and each column represented by a normalized gene coordinate to allow for combining genes of different lengths, and then plotted the sum of these rows using the R programming language [17]. This normalized coordinate system separates each gene into three parts starting from the most 5′ part of the gene to the most 3′ part of the gene: (1) 500 bp upstream of the start of the gene, (2) a normalized region wherein the gene coordinates were mapped to a 1000 bp long window, i.e. the original coordinates were either expanded or compressed to maintain relative distance but would map to within 1000 bp, and (3) 500 bp downstream of the 3′ end point of the gene. The middle window was normalized to ensure that genes of different lengths could be more directly compared with each other. We found that for both previous data and our current DNase I SIM data there is a sharp peak of DHSs located within 500 bp upstream of the transcription start site (TSS) of genes and partway into the 5′ UTR of most genes, with a sharp decline over the length of the gene body, and a final peak that coincides with the transcriptional end-point of genes (Fig. 3), consistent with previous findings [8, 9]. Additionally, these same trends were preserved when employing these direct comparisons using our normalized leaf data (Additional file 3). These findings indicated that, on a global scale, the gene-bias towards DHS gene-localization was preserved across both data sets. We also demonstrated this same localization bias using our root data (Fig. 3), and showed that this general trend is observed across tissue types, indicating a strong bias for DHS peaks occurring around the promoter region, and, to a lesser degree, around transcription termination regions.

#### DHS peaks are highly reproducible

To assess the reproducibility of individual DHSs, we compared the coordinates of DHSs defined by F-Seq [15] and identified those sites that overlapped between our DNase I SIM data sets and those produced using previously published data [8, 9]. In order for a pair of peaks to be considered overlapping between two data sets, at least 80 % of one of the two peaks had to be covered by the corresponding peak. For these analyses, we only used the normalized leaf data in order to provide the most direct comparisons. We found that 70–74 % of all peaks identified in the re-analysis were recapitulated in our normalized data sets (Additional file 4). Additionally we found that when analyzed on a gene-for-gene basis, 90–92 % of genes identified in our re-analysis of this published data were also found to have peaks in our own data sets, providing confidence that the alterations to the original DNase-seq protocol did not affect open chromatin peak identification.

An important point to consider when identifying DHSs is the introduction of background cleavage events. While it is possible to reproduce many of the peaks shown previously, if there is sufficient background noise it could be the case that this is a result of too many false positives contributing to peak agreement. To address this issue, F-Seq generates a background model using a kernel density estimate (kde) of the sequence data for all cleavage events, and then identifies regions that are four standard deviations (by default) above the mean of this kde. To verify that this calculation was estimating a similar level of background cleavage events between our normalized leaf data sets and previously published data, we calculated the percent of all tags that fell within DHSs. We found that ~46 % of our reads fell within identified DHSs in our normalized data sets compared with ~39 % of reads in the re-analyzed leaf data set, indicating that we were generating comparable levels of background cleavage events in our sequenced results.

### Root- and leaf-specific genes show distinct differences in open chromatin

**Fig. 4:**
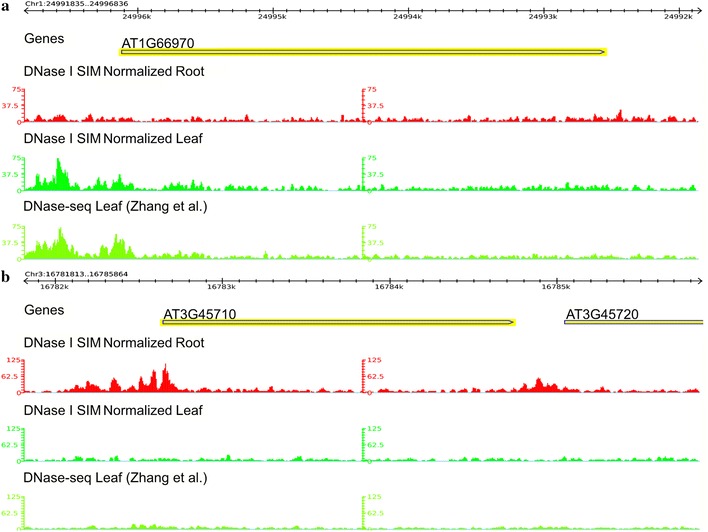
Examples of root- and leaf-specific genes associated with DNase hypersensitive sites. GBrowse screen shots show differential coverage by DNase I SIM reads of root-specific (**a**) and leaf-specific (**b**) genes. For both **a**, **b**, the top track ‘Genes’ identifies the genes that were annotated in a given region, the second track ‘DNase I SIM Normalized Root’ provides a histogram plot of the normalized read coverage found in our root data, the third track ‘DNase I SIM Normalized Leaf’ provides a histogram plot of the normalized read coverage found in our leaf data, and the fourth track ‘DNase-seq Leaf (Zhang et al.)’ provides a histogram plot of the read coverage found in re-analyzed previously published leaf data [9]. Root and leaf data are normalized to have the same read depth as the previously published leaf data

Because of the improvements to the DNase-seq protocol contained in DNase I SIM, we were able to successfully isolate sufficient quantities of genomic DNA to generate a map of DHSs in *Arabidopsis* root tissue. We found that our leaf data DHSs covered ~20,000 genes, while our root data covered ~23,600 genes. Our leaf and root data generated almost identical quantities of uniquely aligned reads, 102 × 10^6^ and 96 × 10^6^ respectively, with a total ~57,000 and ~79,000 DHS identified in leaf and root respectively. In order to highlight some of the differences found, we divided genes with DHSs into three categories: (1) individual genes that were uniquely identified by DHSs in leaf or root (i.e. only leaf or root had a predicted DHS within 500 bp of the gene, or along the gene body), (2) genes identified in both data sets but that showed different DHSs (i.e. individual DHSs in leaf not overlapped by DHSs in root and vice versa), and (3) genes in which DHS sets showed strong overlap between tissue types (i.e. *all* DHSs for a gene overlapped between leaf and root, according to the 80 % coverage requirement for overlap as defined above). Genes that showed strong overlap between DHSs in leaf and root comprised a sizeable category. Of the ~25,200 genes analyzed, ~4000 showed use of strongly overlapping DHS sets. Of the remaining ~21,200 genes, ~14,000 showed DHS peaks in both leaf and root tissues, but differed in where those peaks were used, while only ~1600 and ~5600 genes were unique to leaf and root respectively. Figure 4 shows examples of differential coverage by DHSs of genes predominantly expressed in roots or leaves. In order to show how our data compared to previously published data in *Arabidopsis* [9], we normalized both our root and leaf data to the same depth of sequencing as replicate 1 in [9] in Fig. 4. The strong agreement between our normalized DHS signal and the previously published data gives us further confidence that our approach was successful in reproducing previously published leaf data [9]. The AT3G45710 locus encodes a major facilitator superfamily protein involved in oligopeptide transport. This gene is expressed in root [18]. The distribution of DNase-seq Illumina reads showed that AT3G45710 was clearly associated with open chromatin in root but not in leaf tissues. In contrast, the AT1G66970 locus encoding glycerophosphodiester phosphodiesterase-like protein, which is highly expressed in above ground tissues [19], showed an inverse pattern and was associated with prominent peaks in leaf but not in root tissues. Additional file 5 shows this same comparison with our non-normalized data to highlight the greater depth of sequencing achieved by running our samples on the Illumina HiSeq-2000.

## Discussion

DNase-seq is a technically challenging protocol that provides a great deal of promise in its ability to map DHSs genome-wide in a wide variety of organisms and tissues. Protocol adaptations to tissues with cell walls were critical in expanding the utility of DNase-seq to plants, and have already begun to provide new insights into open chromatin and the epigenetic control of plant genomes [8, 9]. However, because most of the most time-consuming processing steps are performed using agarose gel plugs, tissues with low amounts of DNA or that are particularly recalcitrant due to high levels of cellular debris are prohibitively difficult to study. Using our simplified DNase I SIM protocol, we were able to bypass the gel agarose plugs and provide a method for processing tough plant tissue. More importantly, this new protocol generates sufficient quantities of genomic DNA for sequencing on NextGen sequencing platforms, providing an even greater depth of sequencing than was achieved in the past.

Previous studies were already able to generate DNase-seq data in *Arabidopsis* leaf and flowering tissue [9] and in rice callus and seedling tissue [8], however without the use of transgenics no current studies provide DHS maps for root tissue, a notoriously difficult tissue to process. One aspect of purification approaches that do not use transgenic lines is a realistic requirement for 5–15 g of input tissue depending on tissue type. While this is does not pose a serious limitation in most crop species, it is a feasible but non-negligible quantity in systems such as developing *Arabidopsis* roots. Therefore, if an INTACT transgenic line is available in the whole-organism, tissue, or cell type of interest [12, 13], this should be considered as it provides for an alternative purification strategy that requires less tissue. However, in cases where the production of new transgenic lines of interest requires kanamycin or Basta resistance, or if there is a need for DNase-I studies in mutant lines without an INTACT version, DNase I SIM may provide a potential alternative for generating DHS maps in a given sample of interest.

With our DNase I SIM protocol, we were able to successfully map DHSs genome-wide in *Arabidopsis* root tissue. We found that most DHSs were located near TSSs, in agreement with previous findings [8, 9]. We found that for leaf and root, about 16 % of all genes were associated with strongly overlapping sets of DHSs. Interestingly, most differences found in DHS localization occurred in their distribution within genes, rather than in distinct sets of genes with/without DHSs; however, a number of DHSs did localize to unique sets of genes in both leaf and root tissue.

## Conclusions

In this study, we provide a simplified, more efficient, and time-saving DNase-seq protocol for preparation of genomic DNA libraries for NextGen sequencing. Bypassing the gel-agarose plug processing step allowed a decrease in the length of the protocol by at least 2 days. We successfully applied this protocol to *Arabidopsis* leaf and root tissues, providing for the first time a DHS map of non-transgenic whole root tissue. The data obtained using the modified protocol was consistent with publicly available datasets. We found that 16 % of all genes show strongly overlapping DHS sets between root and leaf tissues, with the largest differences occurring between the location of DHSs within or near a given gene.

## Methods

### Plant material and growth conditions

*Arabidopsis* seeds were surface sterilized in 12 % (w/v) bleach/0.1 % Tween 20 solution and washed extensively with sterile distilled water. Seeds were vernalized for 3 days at 4 °C in water, sown in two parallel rows on MS/agar plates (30 mM sucrose, 4.2 g Murashige and Skoog medium, and 0.8 % Phytagar, pH 5.8) covered with a 100 micron nylon membrane (Genesee Scientific). Seedlings were grown on vertical plates in the Conviron PGR15 growth chamber (12:12 h. light:dark, 21 °C, 50 % humidity, and 250 μmol/m^2^/s light intensity). Roots and leaves of 1 week old seedlings were dissected using surgical blade, flash-frozen in liquid nitrogen, and stored at −80 °C.

### DNase I SIM protocol

Nuclei were isolated from roots and leaves of 1 week old *Arabidopsis* seedlings as described in Additional file 6. Chromatin from isolated nuclei was digested with DNase I, and DNase-seq libraries were prepared as described in Additional file 6. For a full detailed protocol, see Additional file 6. Additional file 7 provides a flowchart that outlines all protocol stages, and shows major differences with the original DNase-seq protocol [6]. Additional file 8 provides a spreadsheet table containing a more detailed view of differences between the DNase I SIM protocol and the protocols in [6] and [10].

### Genome alignment and DHS mapping

All leaf and root tissue reads have been deposited in the SRA [20] under the accession PRJNA285928. DNase-seq data was aligned against the TAIR10 version of the *A. thaliana* genome allowing for up to two mismatches using bowtie [21]. Only those reads that aligned to one genomic locus were used. The peak calling software F-seq [15] was used to identify DHSs, using the aligned reads as input. F-seq version 1.84 was used and ran with a feature length of ‘300’ and only those DHSs that were at least 50 basepairs (bp) long were used for further analysis. In order to identify those DHSs that were shared between data sets, two criteria had to be met: (1) the genome coordinates of the DHS had to overlap, and (2) at least 80 % of one of the two DHSs had to be covered by the other DHS.

### Read depth normalization

For all normalizations, the total number of reads that passed our alignment criteria was calculated and then reads were sampled from our leaf or root data to ensure that the total number of aligned reads in our normalized data set was equal to the number of aligned reads in the previously published data [9]. These normalized alignments were than used to generate DHS maps. This procedure was performed 10 times, and the ranges from these comparisons were noted. For plots, a representative sample of each comparison was provided.

### Analysis of genome-wide DHS distribution

To visualize the distribution of DHSs along the length of the genome, each chromosome was partitioned into non-overlapping bins of equal size. The size of each bin was calculated as the length of the longest chromosome (chromosome 1) divided by 1000. Each subsequent chromosome was then divided into bins of this length to plot the distributions proportionally for each chromosome. The total number of DHSs in each bin was then calculated, and this final value was plotted as a histogram using the R programming language [17].

### Gene DHS matrix distribution

For each gene in a given sample, all of the DHS regions that overlapped the gene and the regions within 500 bp upstream and downstream of the gene were identified. All DHS start and end points were normalized such that: (1) position 1 started 500 bp in the 5′ direction from the gene start, (2) position 2000 was 500 bp in the 3′ direction from the gene start, and (3) positions 501–1500 were the normalized positions that fell within the gene body (e.g. if a DHS ended at 5 bp down from the TSS of a gene that was 500 bp long, the end coordinate would be 510—10 ‘normalized’ bps from the TSS, or position 501). These final normalized coordinate positions were then summed over a matrix, with each position enumerating the number of DHSs that fell within this normalized region, and this total was then plotted using the R programming language [17].

## Additional files

Additional file 1: Distribution of DNase hypersensitive sites along * Arabidopsis* chromosomes. Previously published data was re-analyzed using replicates 1 and 2 from leaf tissue [9]. Approximate boundaries of *Arabidopsis* centromeres [16] are shown in gray. Non-sequenced centromeric gaps are indicated by red circles. Positions of heterochromatic knobs are denoted by violet ellipses. Additional file 2: Distribution of normalized DNase hypersensitive peaks along* Arabidopsis* chromosomes. Previously published data was re-analyzed using replicate 1 from leaf tissue [9] and compared to our leaf data normalized to a similar read depth as replicate 1. Approximate boundaries of *Arabidopsis* centromeres [16] are shown in gray. Non-sequenced centromeric gaps are indicated by red circles. Positions of heterochromatic knobs are denoted by violet ellipses. Additional file 3: Distribution of DNase hypersensitive sites across genes. DHSs across our normalized leaf sample (left), and leaf replicate 1 using previously published data and re-analyzed [9] (right). The x-axis represents the normalized gene length, with positions 1-500 indicating the first 500 bp upstream of the TSS, with the red line indicating the TSS. Positions 501-1500 indicate the gene body, with the green line indicating transcription termination. Positions 1501-2000 indicate the 500 bp downstream of the gene end. Additional file 4: Distribution of overlapping and unique DNase hypersensitive sites in leaf data. Proportion of DNase hypersensitive sites identified as common to both our normalized leaf control and previously published leaf data [9] that we re-analyzed, or that were uniquely identified in each data set. Additional file 5: Examples of root- and leaf-specific genes associated with DNase hypersensitive sites. GBrowse screen shots show differential coverage by DNase-seq reads of root-specific **(A)** and leaf-specific **(B)** genes. For both panels **(A)** and **(B)**, the top track ‘Genes’ identifies the genes that were annotated in a given region, the second track ‘DNase I SIM Root’ provides a histogram plot of the non-normalized read coverage found in our root data, the third track ‘DNase I SIM Leaf’ provides a histogram plot of the non-normalized read coverage found in our leaf data, and the fourth track ‘DNase-seq Leaf (Zhang et al.)’ provides a histogram plot of the read coverage from re-analyzed previously published leaf data [9]. Additional file 6: Detailed DNase-seq protocol. A complete list of reagents and steps for processing plant tissue to prepare DNase-seq without the use of agarose gel plugs. Additional file 7: Experimental flow chart of DNase I SIM protocol and preparation of DNase-seq libraries. Filling-in the DNA ends directly in nuclei (Steps 28-32) avoids embedding and manipulating nuclei in PFGE agarose plugs. This modification increases overall DNA yield and significantly shortens time required for DNA end repair by T4 polymerase as compared to agarose plugs (as described in the original DNase-seq protocol [6]). Library construction steps (shaded box) are described in detail in [6], and step labeling is according to the original protocol provided in [6]. Critical steps are marked by asterisks. * If nuclei yield is lower than 10^6^ nuclei per milliliter and/or nuclei are heavily contaminated with cell debris, do not proceed further. ** Termination of DNase I digestion with EDTA must be conducted rapidly at 4° C and EDTA solution must be removed thoroughly to avoid nuclei lysis and potential inhibition of T4 polymerase activity. *** Concentration of DNA on membrane instead of ethanol precipitation is required to avoid solubility issues of high molecular weight DNA. If the mock sample is even slightly degraded – do not proceed further. Optimal digestion conditions can be assessed by using either 0.9 % SeaKem agarose gels or pulse field gel electrophoresis (PFGE) as described in [6]. It is possible to optimize DNase I concentrations by first using PFGE, and later rapidly assess the digestion quality with pre-determined DNase I concentrations using SeaKem agarose gels. **** Separation of amplified library from linker dimers (steps 39-43 described in [6]) is a critical procedure greatly affecting library quality. Separation of library from dimers in 4%-20% PAGE gel (steps 39-43, [6]) can be substituted by separation in 4.5% NuSieve TBE agarose gels (Lonza) followed by purification of the 86-bp band (containing linkers and insert) using PCR MinElute column (Qiagen). Additional file 8: Detailed list of DNase I SIM changes. Side-by-side spreadsheet of differences at each step found between DNase I SIM and the DNase-seq protocols in [6] and [10].
