# Supplementary figures and images for: Improved DNase-seq protocol facilitates high resolution mapping of DNase I hypersensitive sites in roots in Arabidopsis thaliana
Source: Plant Methods. 2015 Sep 4;11:42. doi: 10.1186/s13007-015-0087-1 (PMC4558764; doi:10.1186/s13007-015-0087-1)

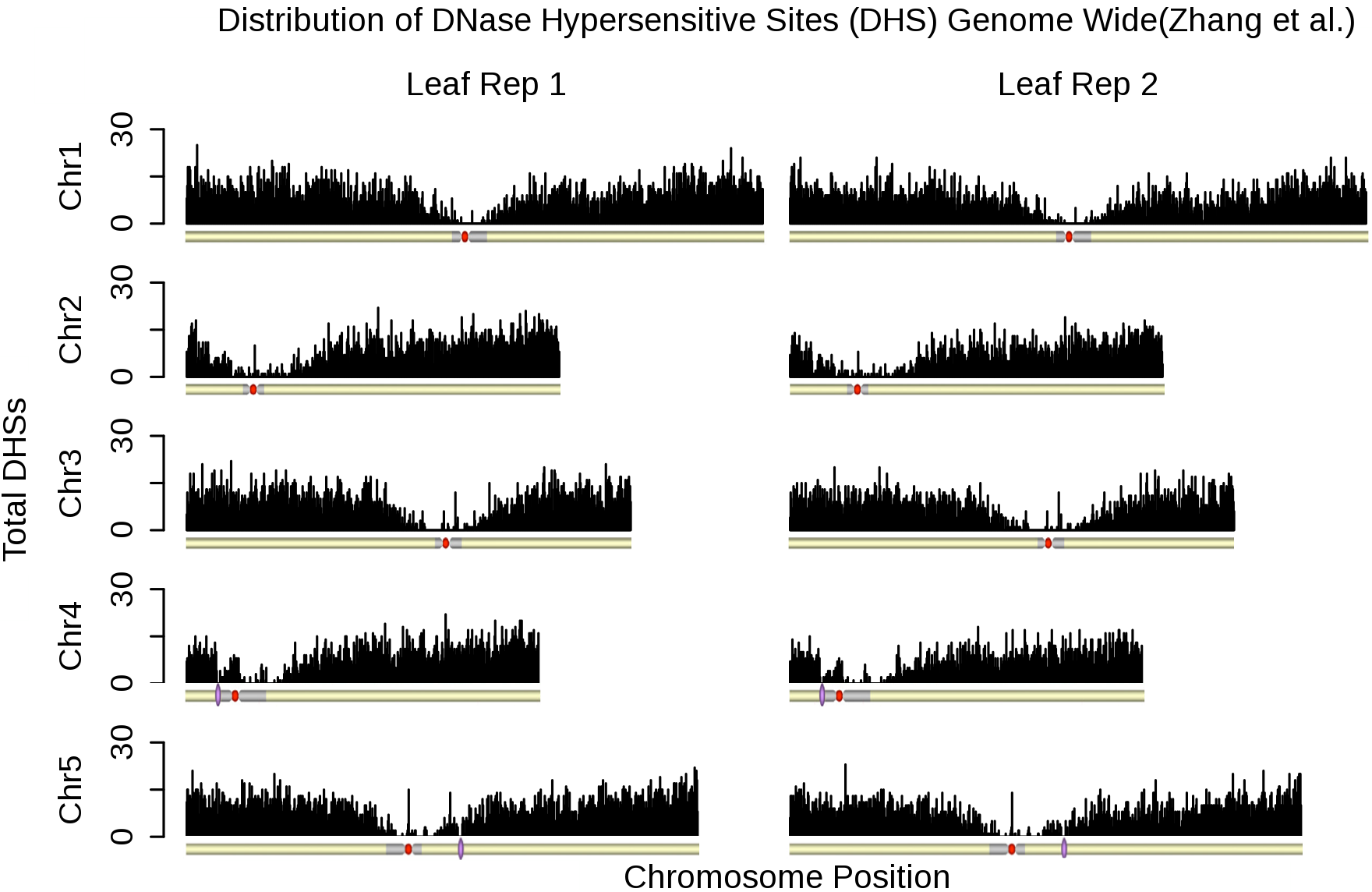

Supplement: Additional file 1: — Distribution of DNase hypersensitive sites along Arabidopsis chromosomes. Previously published data was re-analyzed using replicates 1 and 2 from leaf tissue [9]. Approximate boundaries of Arabidopsis centromeres [16] are shown in gray. Non-sequenced centromeric gaps are indicated by red circles. Positions of heterochromatic knobs are denoted by violet ellipses. [file 13007_2015_87_MOESM1_ESM.png]

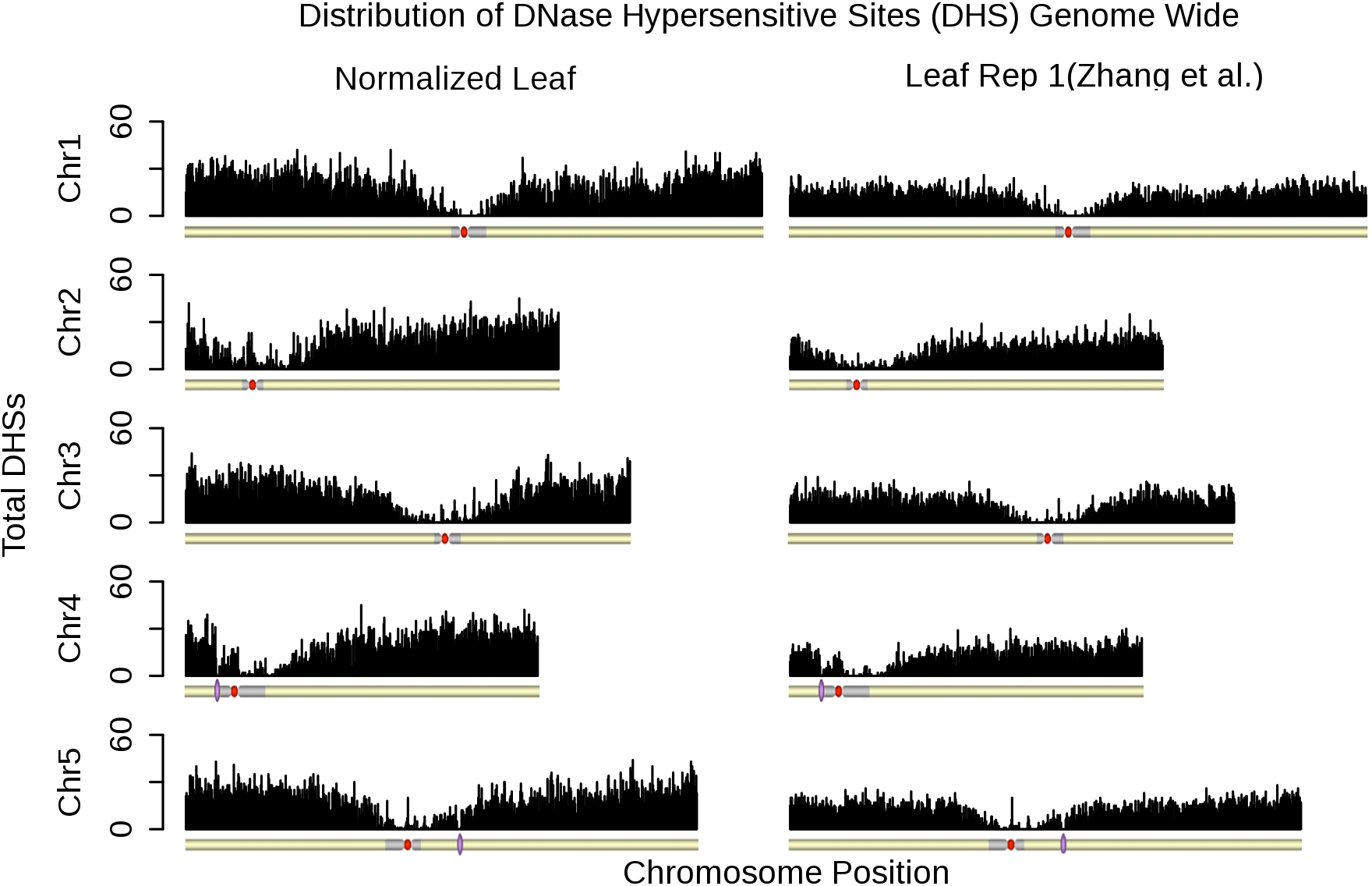

Supplement: Additional file 2: — Distribution of normalized DNase hypersensitive peaks along Arabidopsis chromosomes. Previously published data was re-analyzed using replicate 1 from leaf tissue [9] and compared to our leaf data normalized to a similar read depth as replicate 1. Approximate boundaries of Arabidopsis centromeres [16] are shown in gray. Non-sequenced centromeric gaps are indicated by red circles. Positions of heterochromatic knobs are denoted by violet ellipses. [file 13007_2015_87_MOESM2_ESM.png]

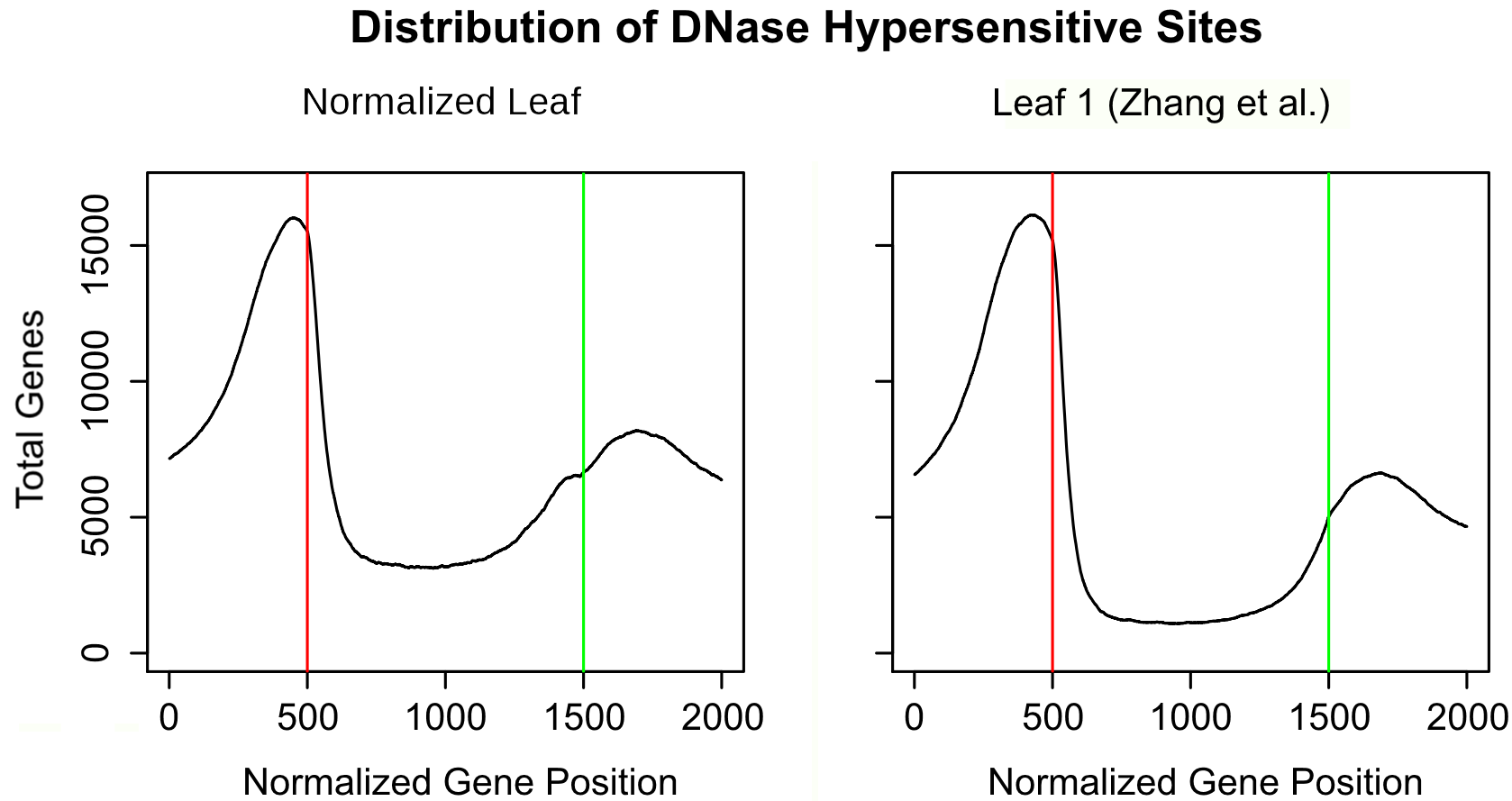

Supplement: Additional file 3: — Distribution of DNase hypersensitive sites across genes. DHSs across our normalized leaf sample (left), and leaf replicate 1 using previously published data and re-analyzed [9] (right). The x-axis represents the normalized gene length, with positions 1-500 indicating the first 500 bp upstream of the TSS, with the red line indicating the TSS. Positions 501-1500 indicate the gene body, with the green line indicating transcription termination. Positions 1501-2000 indicate the 500 bp downstream of the gene end. [file 13007_2015_87_MOESM3_ESM.png]

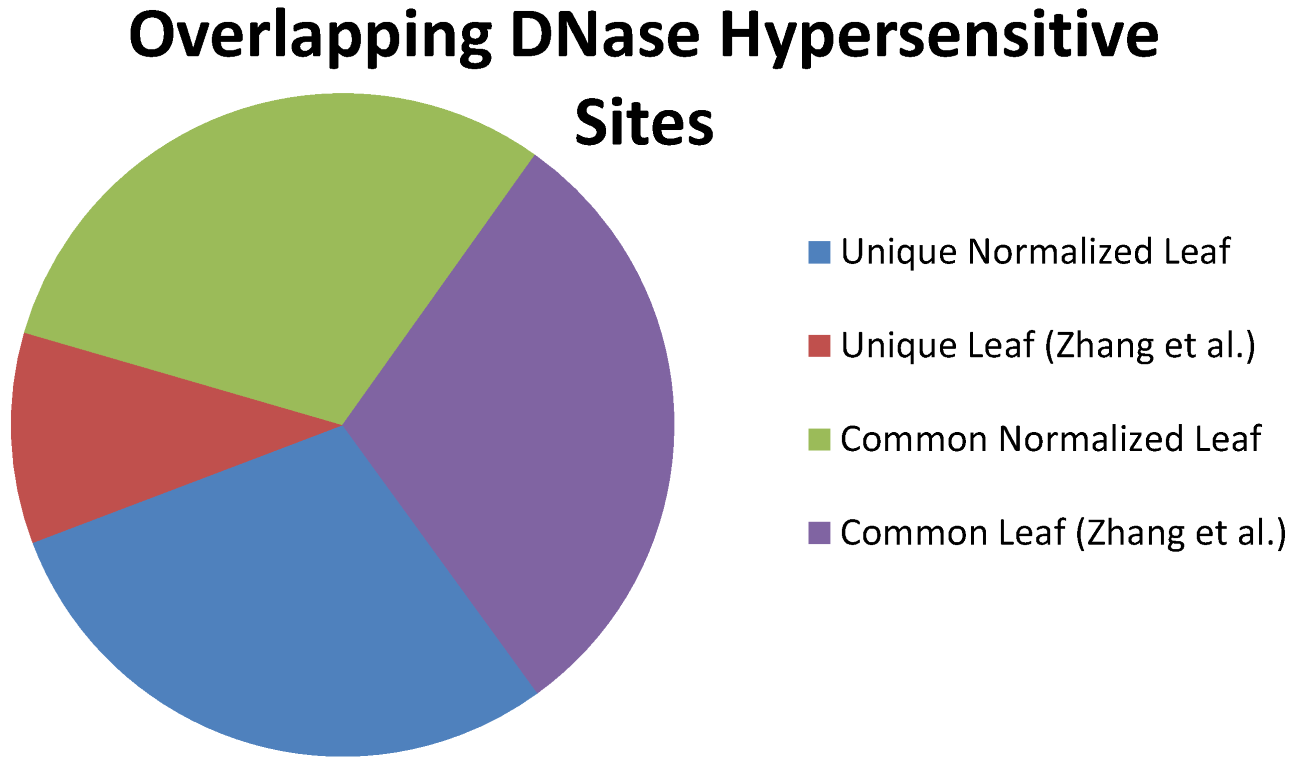

Supplement: Additional file 4: — Distribution of overlapping and unique DNase hypersensitive sites in leaf data. Proportion of DNase hypersensitive sites identified as common to both our normalized leaf control and previously published leaf data [9] that we re-analyzed, or that were uniquely identified in each data set. [file 13007_2015_87_MOESM4_ESM.png]

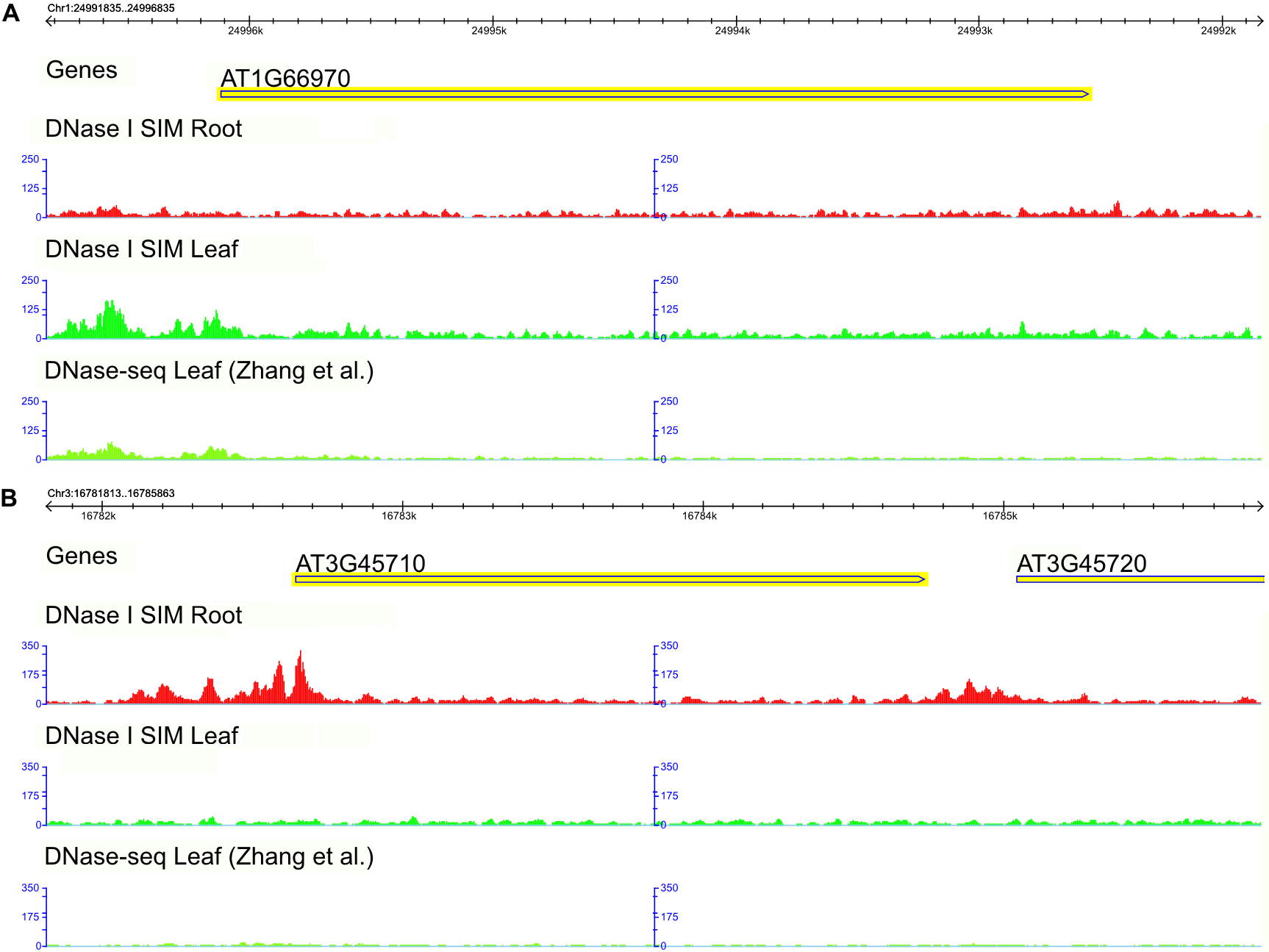

Supplement: Additional file 5: — Examples of root- and leaf-specific genes associated with DNase hypersensitive sites. GBrowse screen shots show differential coverage by DNase-seq reads of root-specific (A) and leaf-specific (B) genes. For both panels (A) and (B), the top track ‘Genes’ identifies the genes that were annotated in a given region, the second track ‘DNase I SIM Root’ provides a histogram plot of the non-normalized read coverage found in our root data, the third track ‘DNase I SIM Leaf’ provides a histogram plot of the non-normalized read coverage found in our leaf data, and the fourth track ‘DNase-seq Leaf (Zhang et al.)’ provides a histogram plot of the read coverage from re-analyzed previously published leaf data [9]. [file 13007_2015_87_MOESM5_ESM.png]

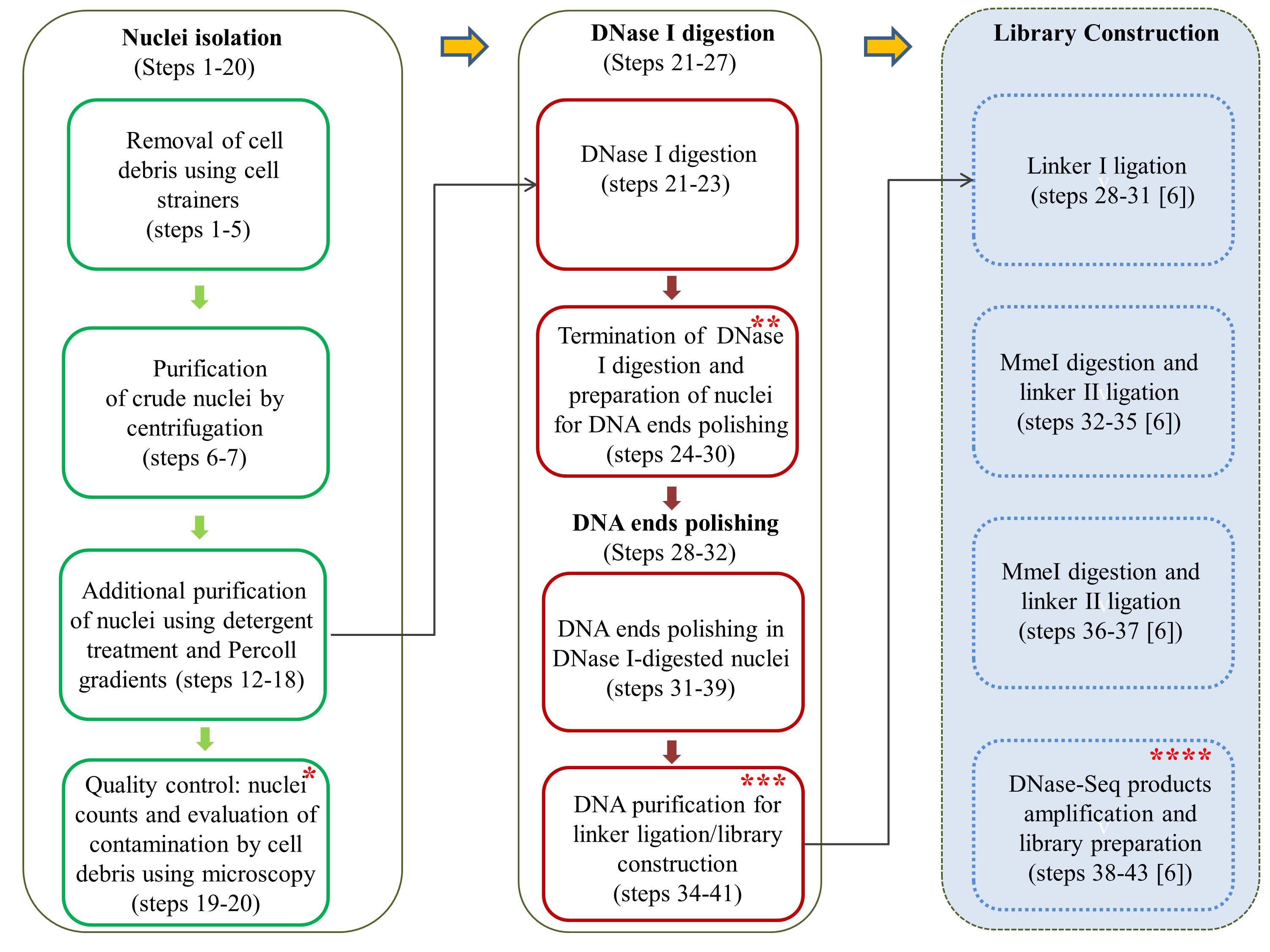

Supplement: Additional file 7: — Experimental flow chart of DNase I SIM protocol and preparation of DNase-seq libraries. Filling-in the DNA ends directly in nuclei (Steps 28-32) avoids embedding and manipulating nuclei in PFGE agarose plugs. This modification increases overall DNA yield and significantly shortens time required for DNA end repair by T4 polymerase as compared to agarose plugs (as described in the original DNase-seq protocol [6]). Library construction steps (shaded box) are described in detail in [6], and step labeling is according to the original protocol provided in [6]. Critical steps are marked by asterisks. * If nuclei yield is lower than 106 nuclei per milliliter and/or nuclei are heavily contaminated with cell debris, do not proceed further. ** Termination of DNase I digestion with EDTA must be conducted rapidly at 4° C and EDTA solution must be removed thoroughly to avoid nuclei lysis and potential inhibition of T4 polymerase activity. *** Concentration of DNA on membrane instead of ethanol precipitation is required to avoid solubility issues of high molecular weight DNA. If the mock sample is even slightly degraded – do not proceed further. Optimal digestion conditions can be assessed by using either 0.9 % SeaKem agarose gels or pulse field gel electrophoresis (PFGE) as described in [6]. It is possible to optimize DNase I concentrations by first using PFGE, and later rapidly assess the digestion quality with pre-determined DNase I concentrations using SeaKem agarose gels. **** Separation of amplified library from linker dimers (steps 39-43 described in [6]) is a critical procedure greatly affecting library quality. Separation of library from dimers in 4%-20% PAGE gel (steps 39-43, [6]) can be substituted by separation in 4.5% NuSieve TBE agarose gels (Lonza) followed by purification of the 86-bp band (containing linkers and insert) using PCR MinElute column (Qiagen). [file 13007_2015_87_MOESM7_ESM.png]
