# Supplementary material for: Improved DNase-seq protocol facilitates high resolution mapping of DNase I hypersensitive sites in roots in Arabidopsis thaliana
Source: Plant Methods. 2015 Sep 4;11:42. doi: 10.1186/s13007-015-0087-1 (PMC4558764; doi:10.1186/s13007-015-0087-1)
Supplement: Additional file 6: — Detailed DNase-seq protocol. A complete list of reagents and steps for processing plant tissue to prepare DNase-seq without the use of agarose gel plugs. [file 13007_2015_87_MOESM6_ESM.docx]

**DNase I SIM Protocol**

**Reagents**

2-(4-amidinophenyl)-1H-indole-6-carboxamidine dihydrochloride (DAPI) (Sigma, cat. No D9542). DAPI stock solution: 0.2 μg/μL, keep in dark.

2-mercaptoethanol (2-ME) (Fisher, cat. No. O3446I-100)

Agarose SeaPlaque GTG (Lonza, cat. No. 182202)

Bovine serum albumin (BSA) (NEB, cat. No. B9000S)

Cell strainers (nylon mesh filters) (Fisher, cat. No. 22363547 (100 μm) and 22363549 (20 μm))

Dithiothreitol (DTT) (Sigma, cat. No. D0632)

Deoxynucleoside triphosphate (dNTPs) set PCR Grade, sodium salt (Roche, cat. No. R11969064001)

DNase Set, RNase-free (Qiagen, 1500 Kunitz units, cat. No. 79254)

2x DNase Stop solution (50 mM Tris-HCl, 100 mM NaCl, 0.1 % SDS, 100 mM EDTA, pH8.0).

Dynabeads M-280 Streptavidin (Invitrogen, cat. No. 11205D)

Ethylenediamine tetraacetic acid (EDTA) (Sigma, cat. No. E7889)

Glycerol (Fisher, cat. No. BP2291)

Glycogen, Ultrapure (Invitrogen, cat. No. 10814010)

Igepal CA630 (Sigma, cat. No. 18896-50)

MgCl_2_ hexahydrate (Fisher, cat. No. BP214-50)

NEBuffer 2 (NEB, cat No. B7002S)

Nitrogen, liquid

Nuclei isolation buffer (NIB): 20 mM Tris-HCl, 25 mM EDTA, 40% (W/V) glycerol pH 7.5 (before using add spermidine to 5 mM, spermine to 0.15 mM, 2-ME to 0.1%, and COMPLETE protease inhibitor).

Percoll-NIB cushion: 20 mM Tris-HCl, 25 mM EDTA, 80% (V/V) Percoll. Add spermidine to 5 mM, spermine to 0.15 mM, 0.1% 2-ME, and adjust pH to7.5 immediately prior to using.

RSB buffer: 20 mM Tris-HCl, 10 mM NaCl, 3 mM MgCl_2_, pH 7.4. Filter sterilize.

Percoll (Sigma, cat. No. P1644)

Potassium chloride (KCl) (Amresco, cat. No. 0395-500g)

Potassium phosphate monobasic (KH_2_PO_4_) (Sigma, cat. No. P8416)

Protease inhibitors COMPLETE, EDTA-free (Roche, cat. No. 11873580001)

Sodium chloride (NaCl) (Fisher, cat. No. BP3581)

Sodium dodecyl sulfate (SDS) (Sigma, cat. No. L3771-25G)

Spermidine (Sigma, cat. No. S0266)

Spermine (MP Biochemicals, cat. No. 100474)

T4 DNA Polymerase (NEB, cat. No. M0203L)

T4 DNA polymerase buffer (final concentrations): 10X NEB Buffer 2 – 1X, dNTPs - 0.3 mM, 100x NEB BSA – 1X.

Tris (Amresco, cat. No. 0497-1kg)

**Materials and equipment**:

Amicon Ultra concentrators (10-kDa cutoff) (Millipore, cat. No. UFC 801024).

Axygen tubes (50 ml) (Axygen, cat. No. SCT-50ml-25S)

Mortar and pestle

Nitex 100 micron nylon membrane (Genesee Scientific, cat. No 57-103) for growing Arabidopsis on vertical plates.

Stainless steel surgical blades

Fluorescent microscope with DAPI filter (e.g., Leica DMRXA)

Miracloth (Fisher, cat. No 50-230-6283)

100, and 40 μm cell strainers (Fisher, cat. No 08-771 and 19 08-771-1, respectively)

Orbital shaker

Pasteur pipettes

Swinging rotor refrigerated centrifuge for 50 ml tubes (e.g., Sorvall Legend R23)

Refrigerated microcentrifuge (e.g., Eppendorf 5424R)

Water bath thermostat

**Isolation of nuclei**

*NOTE*: All solutions should be precooled to 2°C. All manipulations with nuclei should be done at 4°C and the samples should be kept at 4°C or on ice at all times. Add spermidine, spermine, and 2-ME immediately before using solutions.

To isolate nuclei follow steps 1-20

1. Grind 10-15g of one week old Arabidopsis roots in liquid nitrogen to fine powder.
2. Add 25 ml of ice-cold Nuclei Isolation Buffer (NIB) to 10 g of powder (use two 50 ml tubes).
3. Mix using pre-chilled spatula.
4. Keep on ice for 5 min., pipette gently using 5 ml tip with wide bore opening.
5. Filter the slurry subsequently through 100, 40 μm cell strainers (nylon mesh filters), then through a single layer of Miracloth. *Note*: If the slurry is difficult to pass through the insert strainers, a centrifugation at 500 x g for 5 min. in 50 ml tubes could be used.
6. Discard strained cell debris and centrifuge flow-through at 1800 x g at 2°C for 8 min. to pellet nuclei.
7. Decant slowly supernatant.
8. Add 15 ml of NIB supplied with 0.5% Igepal CA630.
9. Carefully re-suspend the nuclei pellet on ice.
10. Centrifuge at 1800 x g at 2°C for 8 min.
11. Repeat steps 8-9.
12. Underlay re-suspended nuclei with 10 ml of 80% Percoll-NIB.
13. Centrifuge at 2200 x g at 2°C for 8 min.
14. Collect nuclei at interphase, dilute in ~ 20 ml of NIB.
15. Centrifuge at 1800 x g at 2°C for 12 min.
16. Re-suspend the nuclei pellet in 15 ml of RSB buffer.
17. Centrifuge at 1100 x g at 2°C for 10 min.
18. Re-suspend in approximately 5 ml of RSB.
19. Add 1 μL of 0.2 μg/μL DAPI to 25 μL of nuclei suspension, keep on ice for 5 min.
20. Count nuclei using UV microscope. At least 10^6^ nuclei per ml are required to continue to DNase I digestion and ends polishing steps. *Note*: Critical checkpoint! If nuclei yield is low and/or if nuclei are heavily contaminated with cell debris and/or chloroplasts (in leaf preparations) do not proceed further.

After counting nuclei proceed immediately to DNase I digestion and ends polishing steps.

**DNase I digestion and DNA ends polishing**

For DNase I digestion follow steps 21-27.

1. Pre-warm for 5 min. at 25°C 6 tubes of 800 μl aliquots of re-suspended nuclei from step 18.
2. Add freshly prepared DNase I (Qiagen cat. # 79254) to a final concentration of 0.0000 (mock control), 0.0002, 0.0005, 0.001, 0.0025, and 0.004 U/ml to each of 800 μl aliquots.
3. Digest for 10 min. at 25°C.
4. Terminate reaction by adding 20 ml of 50 mM EDTA. ** *Critical step!* *Termination of DNase I digestion with EDTA must be conducted rapidly and EDTA solution must be removed thoroughly to avoid nuclei lysis and inhibition of T4 polymerase activity.*
5. Spin at 1100 xg for 10 min.
6. Resuspend in 25 ml RSB.
7. Spin at 1100 x g for 10 min.
8. Resuspend in 10 ml of T4 DNA polymerase buffer.
9. Spin at 1100 xg for 10 min.
10. Resuspend in 2 ml of a T4 DNA polymerase buffer. *Note*: DNA digestion profiles can be evaluated after this step.
11. To polish DNA ends, add 100 ul of T4 DNA Pol (NEB Catalog # M0203L) and Incubate for 3.5 hrs. at RT.
12. Freeze at -20°C.
13. Melt and incubate at RT for 20 min.
14. Stop reaction by addition of 2 ml of 2x DNase stop solution.
15. Add DNase-free Proteinase K to a final concentration of 100 μg/ml, incubate for 2 hrs at 55°C.
16. Add 20 ul of DNase-free RNase A (Fisher), incubate for 30 min at RT.
17. Extract twice with equal volumes of phenol, followed by phenol/chloroform/IAA, and chloroform. To prevent DNA shearing mix by gently inverting tubes and use wide bore tips.
18. Concentrate to 250 μl volume using Amicon Ultra concentrator (10-kDa molecular weight cutoff). *** *Critical step! Concentration of DNA on membrane instead of ethanol precipitation is required to avoid solubility issues of high molecular weight DNA.*
19. Wash concentrated DNA using 1 ml of TE buffer. Concentrate to 250 μl.
20. Assess digestion across the samples using 0.9 % SeaKem agarose (Lonza) in 1x TBE buffer. **** *Critical step!*  *If the mock control sample is even slightly degraded – do not proceed further.*
21. OPTIONAL: digestion across the samples can be assessed using PFGE essentially as described in Song and Crawford (2010) [6].

Proceed with DNase-seq library construction.

1. To ligate adaptors I and II, digest with Mme I, and to construct DNase-seq library follow steps 28-43 described in protocol for mammalian cells in Song and Crawford (2010) [6].
